# Safety and Immunogenicity of sIPV in Healthy Infants Aged 2 Months Following Sequential Immunization Program Combination with bOPV: A Phase 3, Randomized, Blinded, Parallel Positive-Controlled Clinical Trial

**DOI:** 10.3390/vaccines13111094

**Published:** 2025-10-24

**Authors:** Yafei Liu, Xiaodong Liu, Li Zhang, Xianyun Chang, Ping Xiong, Yanxin Guan, Yixin Li, Weiling Zhang, Lili Xuan, Yan Li, Zhifang Ying, Qing Xu

**Affiliations:** 1 Beijing Minhai Biotechnology Co., Ltd., Beijing 102600, China; liuyafei@biominhai.com (Y.L.); changxianyun@biominhai.com (X.C.); guanyanxin@biominhai.com (Y.G.); liyixin@biominhai.com (Y.L.); 2 Shandong Center for Disease Control and Prevention, Jinan 250014, China; liuxd1983@126.com (X.L.); zl9127@163.com (L.Z.); 18615281758@163.com (P.X.); 3 Daiyue District Center for Disease Control and Prevention, Taian 271000, China; zhangweiling2007@163.com (W.Z.); xll1166@126.com (L.X.); dyjxkhb@126.com (Y.L.); 4 National Institute for Food and Drug Control, Beijing 102629, China

**Keywords:** safety, immunogenicity, sIPV, bOPV, sequential immunization

## Abstract

**Objectives**: This phase 3 clinical trial aimed to evaluate the safety and immunogenicity of the Sabin inactivated poliovirus vaccine (sIPV) manufactured by Biominhai in healthy infants following a sequential immunization regimen. **Methods**: A total of 300 healthy infants aged 2 months were randomly divided into the test group (sIPV-sIPV-bOPV) and the control group (wIPV-wIPV-bOPV) according to the ratio of 1:1. Both groups were inoculated under “2IPV + 1bOPV” schedule. Safety was assessed alongside poliovirus antibody levels before and after vaccination. **Results**: The overall incidence of adverse reactions (AEs) in the test and control groups was 44% and 39%, respectively. AEs in both groups primarily occurred following the first dose, with approximately 30% classified as grade 1 in severity. No significant differences were observed between groups regarding the incidence, severity, and symptoms of AEs. Additionally, no vaccine-related serious adverse events (SAEs) were reported. At 30 days after the last dose, the seroconversion rates of neutralizing antibodies against poliovirus types I and III reached 100% in both groups, while type II rates at 99% for the test group and 95% for the control. Notably, the seroconversion rates for all types in the test group were non-inferior to those in the control group. The geometric mean titers (GMTs) of neutralizing antibodies against poliovirus for type I (8622.64 vs. 2687.65), type II (207.73 vs. 54.06), and type III (2121.74 vs. 1699.12) were significantly higher in the test group (*p* < 0.0001 for type I and II; *p* = 0.04 for type III). **Conclusions**: The study concluded that the trial vaccine sIPV following sequential immunization program demonstrates good safety and immunogenicity, showing non-inferiority to the control vaccine.

## 1. Introduction

Poliomyelitis is an intestinal infectious disease caused by the poliovirus, primarily characterized by asymmetric paralysis. The poliovirus has three serotypes (type 1, 2, or 3), each lacking cross-immunity [1]. It predominantly affects children under five years of age and can result in lifelong disability or even death, posing a significant threat to child health [2]. Currently, there are no specific antiviral treatments for poliovirus, vaccination remains the only effective preventive measure [3]. Multiple doses of the polio vaccine can confer long-term protection for children.

In 1988, the World Health Assembly resolved to establish the Global Polio Eradication Initiative (GPEI), which has achieved remarkable success. Since the launch of the initiative, the incidence of wild poliovirus has decreased by over 99%, from an estimated 350,000 cases in more than 125 endemic countries to only five reported cases in 2021 [3,4]. The inactivated poliovirus vaccine (IPV) and bivalent oral poliovirus vaccine (bOPV) have been widely used in China for many years. Currently, China has implemented a sequential vaccination program utilizing two doses of IPV followed by two doses of bOPV. Children receive one dose of IPV at 2 and 3 months of age, and one dose of bOPV at 4 months of age and again at 4 years of age. This strategy aims to build strong immunity against poliovirus through both systemic and mucosal routes. However, due to the potential risks and the higher cost with the production of the Salk strain IPV (wIPV), it is no longer the sole IPV used for polio eradication efforts [2,5]. Consequently, the IPV derived from the Sabin strain (sIPV) has gained popularity in low-income and middle-income countries [5]. It is crucial to determine whether the immunization effects of sIPV in sequential combination with bOPV are equivalent to those of wIPV sequentially with bOPV.

This phase 3, randomized, blinded, parallel positive-controlled clinical trial was performed in Shandong Province, China, beginning in March 2023. The objective was to assess the safety as well as the immunogenicity of sIPV developed by Biominhai (Beijing Minhai Biotechnology Co., Ltd., Beijing, China) within a sequential immunization program, in conjunction with the commercially available bOPV in healthy infants aged 2 months. This study aims to provide a new clinical option for IPV vaccination in infants, contributing to optimized immunization strategies and advancing global poliomyelitis eradication efforts.

## 2. Materials and Methods

### 2.1. Study Design and Population

This was a randomized, blinded, parallel positive-controlled clinical trial conducted in Shandong Province, China, from March 2023 to April 2024. A total of 300 2-month-old healthy infants were enrolled in this trail and randomly assigned to either the test group (sIPV + sIPV + bOPV) or the control group (wIPV + wIPV + bOPV) with a ratio of 1:1. The eligibility criteria were as follows: infants aged 2 months (over 60 days and less than 90 days); infant’s legal guardians agree to sign the informed consent forms voluntarily and are able to comply with the requirements of the clinical trial protocol; and normal armpit temperature. The main exclusion criteria were as follows: (1) preterm infants are delivered before the 37th week of pregnancy; (2) infants with a history of poliomyelitis or polio vaccine immunization; (3) congenital malformations or developmental disorders, genetic defects, severe malnutrition, etc.; (4) infants allergic to any components of the trial vaccine or those with a history of severe allergic reactions to any previous vaccinations; (5) infants with immunodeficiency or receiving immunosuppressive therapy; and (6) infants with a history or family history of convulsions, seizures, encephalopathy, and neurological disorder. And infants who have recently received any other investigational drugs, or have any conditions, as determined by the investigator may affect trial assessments were excluded from our study. The detailed inclusion and exclusion criteria are provided in the Supplementary Materials Table S1. Informed consent was obtained from the legal guardians of all participants prior to enrollment after fully understanding the nature and possible consequences of the study.

This clinical trial was approved by the Vaccine Clinical trials Ethics Committee of the Shandong Provincial Center for Disease Control and Prevention (approval No.: SDJK2022-41-02, approval Date: 11 November 2022). This clinical trial was registered at ClinicalTrials.gov on 30 December 2024 (NCT06752174).

### 2.2. Randomization and Blinding

The study had a randomized, blinded, parallel positive-controlled design. The random number was generated by the sponsor using a random number generator in SAS 9.4 statistical software and was used to number the vaccines. The trial vaccine and control vaccines were placed in a packaging box with the same appearance and only marked with the random number of the vaccine. Infants who meet the inclusion criteria will be assigned the study number in ascending order according to the sequence of enrollment. Subjects were vaccinated with the randomly numbered vaccine corresponding to the study number and were randomly allocated to the test group or control group in a 1:1 ratio. Blinding personnel are not allowed to participate in clinical trials, and they are not allowed to disclose blinding information to anyone participating in the clinical trials.

### 2.3. Vaccines and Vaccinations

Biominhai (Beijing Minhai Biotechnology Co., Ltd., Beijing, China) developed and manufactured the trial vaccine sIPV. Each 0.5 mL dose sIPV contained 15, 45, and 45 D-antigen unit (DU) of poliovirus type 1, type 2, and type 3, respectively. The control vaccine in this study was the wIPV produced by Sanofi Pasteur S.A (Lyon, France). The antigen content of each 0.5 mL dose wIPV was type 1 antigen 40 DU, type 2 antigen 8 DU, and type 3 antigen 32 DU. Both vaccines are delivered intramuscularly. The bOPV produced by BIBP (Beijing Institute of Biological Products Co., Ltd., Beijing, China) is an oral polio vaccine that only contains representative attenuated strains of Type 1 and Type 3 poliovirus. The test group received 2 doses of sIPV and 1 dose of bOPV, and the control group received 2 doses of wIPV and 1 dose of bOPV. The first dose was vaccinated at 2 months of age; each dose was spaced one month apart.

### 2.4. Safety Assessments

Subjects were required to be observed for 30 min in the observation room after each dose of the vaccination. Researchers must record any adverse events (AEs) occurring within 30 min for each infant. All solicited local or systemic AEs and unsolicited AEs occurring from 30 min to 30 days post each vaccination were recorded by the legal guardians of all infants in a diary card. Solicited local AEs include pain, swelling, induration, erythema, rash, and cellulitis. Solicited systemic AEs include fever, diarrhea, anorexia, nausea, new-onset convulsions, cough, mucocutaneous abnormality, irritation, and acute allergic reaction. Within 30 days after each injection, researchers conducted a follow-up visit to verify the safety records. Any serious AEs (SAEs) were also monitored and collected throughout the 6-month follow-up period following the last vaccination. The severity of AEs was graded according to the clinical trial guidelines of preventive vaccines issued by the China Medical Products Administration [6]. The grading criteria were as follows: Grade 1 (Mild): Transient (≤48 h) or mild discomfort that does not affect daily activities and does not require treatment. Grade 2 (Moderate): Mild to moderate restriction of activity, potentially requiring medical consultation but not necessitating or requiring only minimal treatment. Grade 3 (Severe): Significant restriction of activity, requiring medical consultation and treatment, possibly necessitating hospitalization. Grade 4 (Life-threatening): Potentially life-threatening, with severe restriction of activity, requiring intensive monitoring and treatment. Grade 5 (Death). The detailed grading criteria for AEs are provided in the Supplementary Materials Tables S2–S4.

### 2.5. Immunogenicity Assessments

Sera (approximately 2.0 mL) from all infants were collected prior to immunization and on the 30th day after the last immunization. The serum samples were separated on the same day of collection, and they should be promptly stored at −20 °C until measured. The anti–poliovirus neutralizing antibody titer in serum was detected by microneutralization assay recommended by WHO at the National Institutes for Food and Drug Control, China (NIFDC) [7,8]. The primary endpoint was the seroconversion rate of antibodies on day 30 after the final dose. The secondary endpoints were the seropositive rate of antibodies and Geometric mean titer (GMT). The neutralizing antibody against poliovirus types I, II, and III titer ≥1:8 was considered to be seropositive. Seroconversion was defined as negative antibody levels before immunization and positive levels after immunization, or a fourfold increase in antibody levels after immunization if the antibodies were positive before immunization. For infants with maternal antibodies before vaccination, the calculation of the neutralizing antibody titer was adjusted for maternal antibodies, assuming an exponential decay model with a half-life of 28 days.

### 2.6. Statistical Analysis

All statistical analyses were conducted using SAS Version 9.4. Pearson’s chi-squared test was used to compare the incidence of AEs, seropositive rates, and seroconversion rates between the two groups. If the sample size was less than 40, Fisher’s exact test was used to compare the differences between the groups. The GMT of neutralizing antibody was analyzed using either the *t*-test, applied when the two independent groups had equal variances, or the *t*’-test, used when the variances were unequal. The confidence interval (95% CI) for the difference in seroconversion rates between the test and control groups was calculated. Non-inferiority was defined as the lower bound of the 95% CI for the difference between groups exceeded the inferiority margin of −10%. *p* ≤ 0.05 was considered statistically significant in this study.

## 3. Results

### 3.1. Study Population

**Figure 1:**
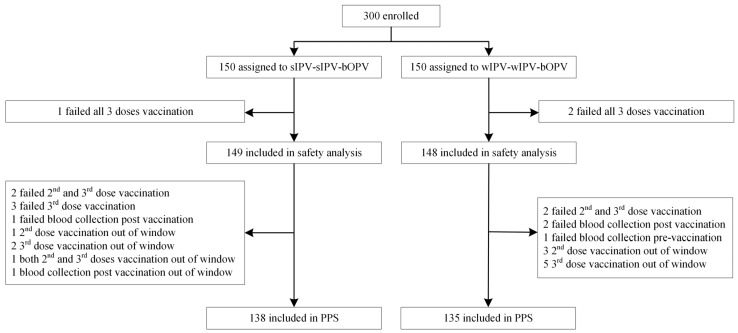
Profile of the study. PPS, per-protocol analysis set for immunogenicity analysis. Immunogenicity analysis was performed in susceptible subjects of PPS. Susceptible subjects were defined as having a pre-immune antibody titer of ≤1:8.

A total of 300 healthy infants were enrolled in this trial and were randomly divided into two groups (150 infants per group). The safety analysis set (SS) comprised 297 subjects who received the first vaccination, including 149 subjects from the test group (sIPV-sIPV-bOPV) and 148 subjects from the control group (wIPV-wIPV-bOPV). Due to 7 subjects (5 in test group and 2 in control group) failing vaccination, 4 subjects (1 in test group and 3 in control group) failing blood collection, 12 subjects (4 in test group and 8 in control group) being vaccinated out of window, and 1 subject in the test group having blood collection post vaccination out of window, 273 subjects ultimately entered the per-protocol set (PPS) for immunogenicity analysis, including 138 subjects from the test group, and 135 subjects from the control group (Figure 1).

At enrollment, the mean age was 2.25 months in sIPV-sIPV-bOPV and 2.30 months in wIPV-wIPV-bOPV. The pre-immune GMTs for type 1 in the test and control group were 2.62 and 2.70, respectively; for type 2, they were 2.59, and 2.44, for type 3, they were 3.20 and 3.34. The pre-immune susceptible subject rates for type 1 were 95% in the test (131/138) and control group (128/135), respectively; for type 2, they were 97% (134/138) and 99% (133/135), and for type 3, they were 100% in both groups. The detailed baseline characteristics are provided in Table 1. There were no significant differences regarding age, sex, axillary temperature, BMI, antibody GMT, and susceptible subject rates of the three polio types between the two groups prior to immunization (Table 1).

### 3.2. Safety

**Figure 2:**
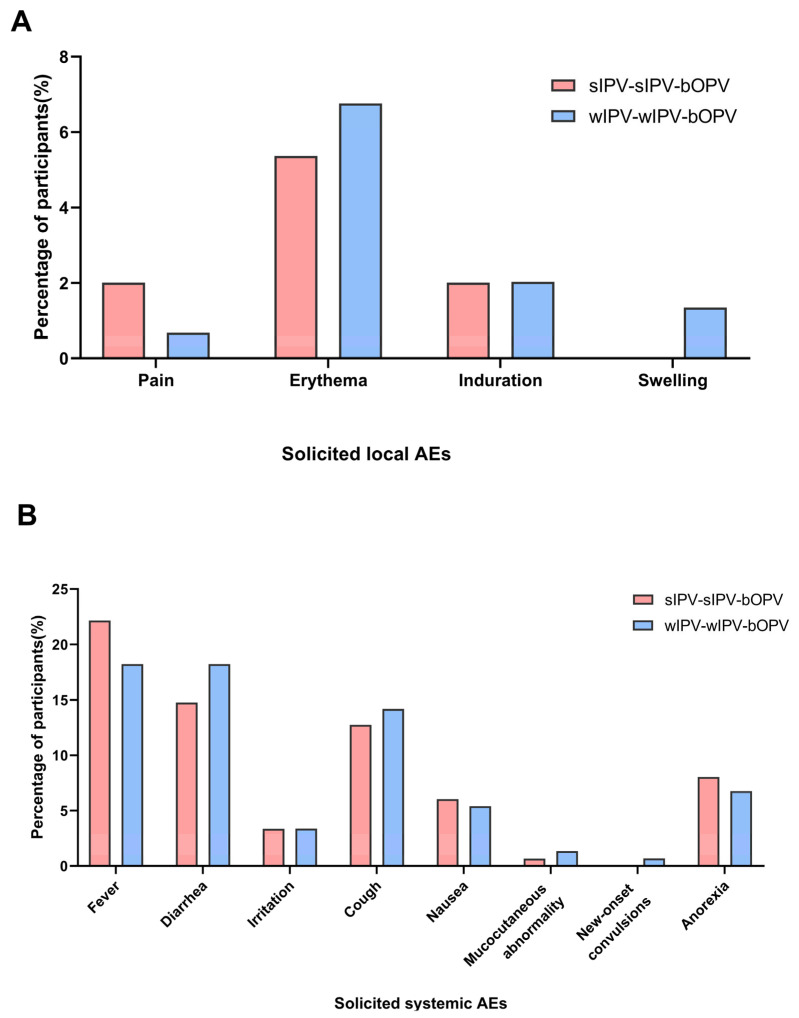
Percentage of infants who experienced solicited local AEs (**A**) or solicited systemic AEs (**B**) from the first dose through 30 days after the final dose.

Table 2 presents the incidence of AEs among the subjects. From the first dose to 30 days after the last dose, the incidence of total AEs associated with vaccination was 44% in the test group and 39% in the control group. In the test group, 5% of the subjects experienced solicited local AEs, with erythema being the most frequently observed symptom (Figure 2A). Additionally, 42% of the subjects reported solicited systemic AEs, primarily characterized by fever, diarrhea, and cough (Figure 2B). In contrast, the control group reported a 7% incidence of solicited local AEs and a 36% incidence of solicited systemic AEs. Notably, the majority of reported AEs were classified as Grade 1 in severity. Statistical analysis revealed no significant differences between the two groups regarding the incidence and severity of AEs. Furthermore, the incidence of AEs symptoms did not demonstrate statistically significant differences, and neither group reported unsolicited AEs.

Table 3 illustrates the incidence of AEs after each vaccination in both the test and control groups. AEs primarily occurred after the first immunization, with incidences of 23% and 22% in the test and control groups, respectively. There were no statistically significant differences between the groups for AEs following each dose. Within six months following the initial vaccination, the incidence of SAEs was 7% (11 subjects) in the test group and 10% (15 subjects) in the control group, with no statistically significant differences between the groups. Importantly, all subjects with SAEs had recovered, and the investigator concluded that all SAEs were unrelated to the vaccination.

### 3.3. Immunogenicity

The immunogenicity analysis was conducted in susceptible subjects of PPS. Susceptible subjects were defined as having a pre-immune antibody titer of ≤1:8. At 30 days post the final dose, the seroconversion rates for type II poliovirus neutralizing antibodies in the test and control groups were 99% and 95%, respectively, while the seroconversion rates for types I and III antibodies were both 100%. Comparison of the seroconversion rates between the groups showed no statistically significant differences. The seropositive rate results were consistent with the seroconversion rates. Furthermore, a non-inferiority analysis of the seroconversion rates indicated that the test group was non-inferior to the control group for all antibody types. The GMTs of type I poliovirus neutralizing antibodies in the test and control groups were 8622.64 and 2687.65, respectively; the GMTs for type II antibodies were 207.73 and 54.06, and for type III antibodies, they were 2121.74 and 1699.12, respectively. The GMTs of all antibody types in the test group were significantly higher than those in the control group (*p* < 0.0001, *p* < 0.0001, *p* = 0.04) (Table 4).

## 4. Discussion

Poliomyelitis primarily occurs through fecal-oral and oral-oral routes, and it can invade the nervous system, leading to paralysis [1]. Relevant studies indicate that circulating antibody generated by multiple-dose immunization persists for decades and possibly for life [9,10,11]. The available polio vaccines include the oral polio vaccine (OPV) and the IPV. OPV contains attenuated live poliovirus. Administration of OPV is similar to natural exposure to the poliovirus, inducing both humoral and mucosal immunity. However, due to individual differences and immune deficiencies among recipients, a small number of children may develop vaccine-associated paralytic poliomyelitis (VAPP) after receiving OPV or coming into contact with others who have received OPV [2,12]. The clinical manifestations of VAPP closely resemble those of polio [13].

The GPEI has made significant progress, achieving the certification of the global eradication of wild poliovirus type 2 (WPV2) in 2015. In 2016, a global switch was implemented, transitioning from the trivalent oral polio vaccine (tOPV) to the bOPV, which contains only types 1 and 3 [2]. This switch was supported by the introduction of at least one dose of IPV to mitigate the risk of VAPP while maintaining the high levels of intestinal mucosal immunity induced by OPV [2]. The transition to bOPV and the use of IPV reflect the evolving understanding of how best to balance the eradication of polio with the minimization of risks associated with vaccination. IPV is prepared through cell culture, purification, and formalin inactivation of the three poliovirus serotypes. Currently available IPVs include sIPV, which is produced using the Sabin attenuated strain, and wIPV, which is derived from wild poliovirus (WPV). Utilizing attenuated Sabin strains instead of wild strains enhances safety during vaccine production [14].

Since January 2020, the routine immunization schedule for polio vaccines in China was adjusted from 1 dose of IPV along with 3 doses of bOPV to 2 doses of IPV and 2 doses of bOPV. Infants receive 1 dose of IPV at 2 and 3 months of age and 1 dose of bOPV at 4 months and again at 4 years. Based on the safety profile of sIPV and the market demand for polio vaccines, sIPV developed by Biominhai conducted polio vaccination policy with bOPV in the phase 3 clinical trial aimed at obtaining marketing authorization in China. In general, this study assessed the safety and immunogenicity of the sIPV produced by Biominhai following the “2IPV + 1bOPV” sequential immunization schedule in healthy infants at 2 months of age by comparing them with the wIPV produced by Sanofi Pasteur.

The trial vaccine exhibited good tolerance and comparable safety to other IPV. The most frequently reported solicited local AEs included erythema, induration, and pain, while solicited systemic AEs encompassed fever, diarrhea, and cough, with the majority classified as Grade 1, and no Grade 3 or higher reactions were reported. No significant difference in safety results between the test and control groups. During the six-month long-term follow-up period following the last dose, there was no SAEs related to the vaccination. The AE symptoms were similar to other studies on commercial polio vaccines, indicating the good safety of the trial vaccine [15,16]. Furthermore, the candidate sIPV demonstrated excellent immunogenicity, achieving 100% seroconversion rates for antibodies against poliovirus types I and III, and 99.25% for type II on 30 days post the final dose. Notably, the seroconversion rates for all types were non-inferior to those of the control group. Additionally, the GMTs of neutralizing antibodies for poliovirus types I, II, and III in the test group were significantly elevated compared to those in the control group (*p* < 0.0001, *p* < 0.0001, *p* = 0.04). The D-antigen of type 1 in sIPV (15 DU) is lower than in wIPV (40 DU), yet the GMT of neutralizing antibodies remains significantly higher in sIPV compared to wIPV (8622.64 vs. 2687.65). The D-antigen of type 2 in wIPV (8 DU) is lower than that in sIPV (45 DU), which may contribute to the differences in type 2 GMTs (207.73 vs. 54.06). Despite this, the overall immune response induced by sIPV was robust, and the vaccine demonstrated its ability to generate high levels of neutralizing antibodies against all three types of poliovirus. The successful evaluation of sIPV following the “2IPV + 1bOPV” sequential immunization schedule highlights its potential as a viable option for poliovirus prevention. This trial provides important evidence that sIPV can elicit strong immune responses while maintaining a high safety profile. It is important to note that the effects of maternal antibodies were accounted for in this study, and the immunogenicity results remained consistent before and after maternal antibody correction.

Nonetheless, several areas can be further explored. This clinical trial was performed in healthy infants aged 2 months, explicitly excluding premature infants and immunocompromised individuals, which introduces certain limitations to the findings. Additionally, since IPVs are frequently co-administered with other vaccines, future research will assess the safety and immunogenicity of the trial vaccine when combined with vaccines such as diphtheria–tetanus–cellular pertussis (DTaP), Hib, and hepatitis B, if necessary.

## 5. Conclusions

The trial vaccine sIPV, developed by Biominhai (Beijing Minhai Biotechnology Co., Ltd., Beijing, China), demonstrated non-inferiority to the commercially available wIPV in terms of immunogenicity and safety when administered under the “2IPV + 1bOPV” sequential vaccination regimen. In conclusion, the trial vaccine exhibited favorable safety and immunogenicity following the sequential immunization schedule.

## Figures and Tables

**Table 1:** Baseline characteristics.

	sIPV-sIPV-bOPV (*n* = 138)	wIPV-wIPV-bOPV (*n* = 135)	*p* Value
Age, mean ± SD (months)	2.25 ± 0.23	2.30 ± 0.25	0.13
sex, *n* (%)			
Male: *n* (%)	65 (47)	66 (49)	0.77
Female: *n* (%)	73 (53)	69 (51)	
axillary temperature at enrolment, mean ± SD (°C)	36.84 ± 0.16	36.83 ± 0.16	0.88
BMI, mean ± SD (kg/m^2^)	16.08 ± 1.52	15.88 ± 1.47	0.27
Type 1			
Pre-immune GMT (95% CI)	2.62 ± 1.87 (2.35~2.92)	2.70 ± 1.94 (2.41~3.03)	0.70
Pre-immune susceptible subjects: *n* (%)	131 (95)	128 (95)	0.97
Type 2			
Pre-immune GMT (95% CI)	2.59 ± 1.93 (2.31~2.89)	2.44 ± 1.97 (2.17~2.74)	0.48
Pre-immune susceptible subjects: *n* (%)	134 (97)	133 (99)	0.68
Type 3			
Pre-immune GMT (95% CI)	3.20 ± 1.73 (2.91~3.51)	3.34 ± 1.65 (3.06~3.63)	0.50
Pre-immune susceptible subjects: *n* (%)	138 (100)	135 (100)	/

SD, standard deviation; Susceptible subjects, the pre-immune antibody titer of the individual is ≤1:8.

**Table 2:** Incidence of overall AEs from the first dose until 30 days after the final dose of vaccine in this study.

	sIPV-sIPV-bOPV (*n* = 149)	wIPV-wIPV-bOPV (*n* = 148)	*p* Value
Total AEs	65 (44%)	57 (39%)	0.37
Grade 1	48 (32%)	44 (30%)	0.64
Grade 2	29 (19%)	29 (20%)	0.98
Grade 3	7 (5%)	3 (2%)	0.34
Solicited local AEs	8 (5%)	11 (7%)	0.47
Grade 1	5 (3%)	9 (6%)	0.27
Grade 2	2 (1%)	2 (1%)	1.00
Grade 3	1 (0.67%)	0 (0.00%)	1.00
Solicited systemic AEs	62 (42%)	53 (36%)	0.30
Grade 1	32 (21%)	24 (16%)	0.25
Grade 2	24 (16%)	26 (18%)	0.74
Grade 3	6 (4%)	3 (2%)	0.50

Grade 1 is mild; Grade 2 is moderate; Grade 3 is severe.

**Table 3:** Incidence of AEs of each injection.

	sIPV-sIPV-bOPV	wIPV-wIPV-bOPV	*p* Value
First injection, n/N_SS1_	35/149 (23%)	33/148 (22%)	0.81
Second injection, n/N_SS2_	22/147 (15%)	24/146 (16%)	0.73
Third injection, n/N_SS3_	19/144 (13%)	24/146 (16%)	0.44

N_SS1_/N_SS2_/N_SS3_, the number of infants who received the first/second/third injection.

**Table 4:** Immunogenicity analysis in susceptible subjects of sIPV-sIPV-bOPV and wIPV-wIPV-bOPV.

	sIPV-sIPV-bOPV	wIPV-wIPV-bOPV	*p* Value	Difference% (95% CI)	Non-Inferiority * Yes/No
Type 1					
Seropositive (%) (95% CI)	100 (97.22~100.00)	100 (97.16~100.00)	/		
Seroconversion (%) (95% CI)	100 (97.22~100.00)	100 (97.16~100.00)	/	0.00 (−2.85~2.91)	Yes
GMT (95% CI)	8622.64 ± 2.08 (7594.47~9790.01)	2687.65 ± 1.97 (2386.47~3026.83)	<0.0001		
Type 2					
Seropositive (%) (95% CI)	99 (95.91~99.98)	95 (90.44~98.33)	0.07		
Seroconversion (%) (95% CI)	99 (95.91~99.98)	95 (90.44~98.33)	0.07	3.77 (−0.05~7.58)	Yes
GMT (95% CI)	207.73 ± 3.05 (171.67~251.37)	54.06 ± 2.93 (44.96~65.00)	<0.0001		
Type 3					
Seropositive (%) (95% CI)	100 (97.36~100.00)	100 (97.30~100.00)	/		
Seroconversion (%) (95% CI)	100 (97.36~100.00)	100 (97.30~100.00)	/	0.00 (−2.71~2.77)	Yes
GMT (95% CI)	2121.74 ± 2.59 (1807.25~2490.95)	1699.12 ± 2.26 (1479.26~1951.65)	0.04		

* Non-inferiority was achieved if the lower limit of the 95% CI of the difference (sIPV-sIPV-bOPV)–(wIPV-wIPV-bOPV) for the seroconversion was >−10%.

## Data Availability

The original contributions presented in this study are included in the article. Further inquiries can be directed to the corresponding authors.

## Supplementary Materials

The following supporting information can be downloaded at: https://www.mdpi.com/article/10.3390/vaccines13111094/s1, Table S1: Inclusion and Exclusion Criteria; Table S2: Grading scale for adverse events at the injection site (local); Table S3: Vital signs grading scale; Table S4: Grading scale for adverse events at non-injection sites (systemic).
