# Supplementary material for: Safety and Immunogenicity of sIPV in Healthy Infants Aged 2 Months Following Sequential Immunization Program Combination with bOPV: A Phase 3, Randomized, Blinded, Parallel Positive-Controlled Clinical Trial
Source: Vaccines (Basel). 2025 Oct 24;13(11):1094. doi: 10.3390/vaccines13111094 (PMC12656725; doi:10.3390/vaccines13111094)
Supplement: Supplementary file 1 [file vaccines-13-01094-s001.zip › vaccines-3911325-supplementary.pdf]

Table S1. Inclusion and Exclusion Criteria

Inclusion Criteria:

1. Healthy permanent residents aged 2 months (over 60 days and less than 90 days);
2. Infant's legal guardians agree to sign the informed consent forms voluntarily;
3. Infant's legal guardians are able to comply with the requirements of the clinical trial protocol;
4. Armpit temperature ≤ 37.0 ℃

Exclusion Criteria:

1. Preterm infants (delivery before the 37th week of pregnancy).
2. Previous vaccination against polio.
3. Congenital malformations or developmental disorders, genetic defects, severe malnutrition, etc.
4. History of polio.
5. History or family history of convulsions, seizures, encephalopathy, and neurological disorder.
6. History of a serious reaction to any prior vaccination or known hypersensitivity to any component of the investigational vaccine.
7. Individuals with immunodeficiency or receiving immunosuppression therapy.
8. Disturbance of coagulation diagnosed by doctor (e.g., coagulation factors deficiency, coagulation disease, platelet abnormality) or obvious bruising or coagulation disorder.
9. Known or suspected concomitant diseases include: respiratory disease, acute infection or active chronic disease, cardiovascular disease, liver and kidney disease, skin disease, mother with HIV infection.
10. Administration of blood or blood-related products or immunoglobulins (hepatitis B immunoglobulins is acceptable).
11. History of administration of live attenuated vaccines within 14 days.
12. History of administration of subunit or inactivated vaccines within 7 days.
13. Individuals with any acute diseases within 7 days, receiving antibiotics or antiviral therapy.
14. Fever within 3 days (Armpit temperature ≥38.0 ℃).
15. Use of any investigational product recently, or have any conditions that the investigator believes may affect the evaluation of the trial.

Grading criteria for AEs:

Table S2. Grading scale for adverse events at the injection site (local)

Symptoms/ Signs	Grade 1	Grade 2	Grade 3	Grade 4
Pain	Avoidance or withdrawal upon contact or	Crying or fussing upon contact or	Persistent crying that cannot be	Requires emergency care or

Symptoms/ Signs	Grade 1	Grade 2	Grade 3	Grade 4
	touch	touch, but can be soothed	soothed	hospitalization
Induration*, Swelling**#	Diameter < 2.5 cm	Diameter ≥ 2.5 cm, and the area < 50% of the vaccinated limb (anatomical site of the limb, such as the upper arm or thigh)	Area ≥ 50% of the vaccinated limb, or ulceration, secondary infection, phlebitis, or wound drainage	Abscess, exfoliative dermatitis, dermal or deep tissue necrosis
Rash*, Erythema**#	Diameter < 2.5 cm	Diameter ≥ 2.5 cm, and the area < 50% of the vaccinated limb (referring to the anatomical site of the limb, such as the upper arm or thigh)	Area ≥ 50% of the vaccinated limb, or ulceration, secondary infection, phlebitis, or wound drainage	Abscess, exfoliative dermatitis, dermal or deep tissue necrosis
Cellulitis	NA	Non-injection treatment is required, such as oral antibacterial, antifungal, or antiviral drug therapy.	Intravenous therapy is required, such as intravenous antibacterial, antifungal, or antiviral drug treatment.	Sepsis or tissue necrosis, etc.

Note: *In addition to grading based on direct measurement of the diameter, the progression of the measurement results should also be documented. **The maximum measured diameter or area should be used. #The evaluation and grading of induration, swelling, rash, and erythema should be based on functional grading and actual measurement results, with the higher grade indicator selected.

Table S3. Vital signs grading scale

Signs	Grade 1	Grade 2	Grade 3	Grade 4
**Fever* [axillary temperature,℃**	37.5~<38.0	38.0~<39.5	≥39.5	≥39.5, Persisting for more than 5

]				days

Note: *In China, axillary temperature is commonly used, and when necessary, it is converted to oral and rectal temperatures. Typically, oral temperature = axillary temperature + 0.2 °C; rectal temperature = axillary temperature + (0.3~0.5 °C). In cases of persistent high fever, the cause of the fever should be promptly determined.

Table S4. Grading scale for adverse events at non-injection sites (systemic)

Organ system symptoms/Signs	Grade 1	Grade 2	Grade 3	Grade 4
**Gastrointestinal System**				
**Diarrhea**	Mild or transient, 3-4 times per day, abnormal stool consistency, or mild diarrhea lasting less than 1 week	Moderate or persistent, 5-7 times per day, abnormal stool consistency, or diarrhea lasting more than 1 week.	>7 times per day, abnormal stool consistency, or hemorrhagic diarrhea, orthostatic hypotension, electrolyte imbalance, requiring intravenous fluid administration >2L.	Hypotensive shock, requiring hospitalization
**Anorexia**	Decreased appetite without reduction in food intake.	Decreased appetite, reduced food intake, but no significant weight loss.	Decreased appetite with significant weight loss	Requires intervention measures (e.g., gastric tube feeding, parenteral nutrition)
**Nausea**	1-2 times per 24 h without affecting activity.	3-5 times per 24 h or activity limited.	More than 6 times within 24 hours or requires intravenous fluid replacement.	Requires hospitalization or alternative nutritional support due to hypotensive shock
**Nervous System**				
**New-onset convulsions**	Seizure duration < 5 minutes, and	Seizure duration ≥ 5 to < 20 minutes, and	Seizure duration ≥ 20 minutes or	Prolonged and multiple seizures (e.g.,

Organ system symptoms/Signs	Grade 1	Grade 2	Grade 3	Grade 4
	postictal state < 24 h	postictal state < 24 h	postictal state > 24 h	status epilepticus) or difficult to control (e.g., refractory epilepsy)
**Respiratory System**				
**Cough**	Transient, no treatment required.	Persistent cough, treatment effective.	Paroxysmal cough, treatment uncontrolled	Emergency or hospitalization
**Skin and subcutaneous tissue**				
**Mucocutaneous abnormality**	Erythema / Pruritus / Color changes.	Diffuse rash / Maculopapular rash / Dryness / Desquamation.	Vesicular / Exudative / Desquamation / Ulceration	Exfoliative dermatitis involving mucous membranes, or erythema multiforme, or suspected Stevens-Johnson syndrome
**Nervous system**				
**Irritation or suppression**	Mild irritation or suppression	Irritability or drowsiness	Inconsolable or decreased responsiveness	NA
**Immune system**				
**Acute allergic reaction** **	Local urticaria (vesicles), no treatment required	Local urticaria, treatment required, or mild angioedema, no treatment required	Extensive urticaria or angioedema requiring treatment, or mild bronchospasm	Anaphylactic shock or life-threatening bronchospasm or laryngeal edema

Note: **Type I hypersensitivity reaction.
